# Supplementary material for: Origins and Bioactivities of Natural Compounds Derived from Marine Ascidians and Their Symbionts
Source: Mar Drugs. 2019 Nov 28;17(12):670. doi: 10.3390/md17120670 (PMC6950356; doi:10.3390/md17120670)
Supplement: Supplementary file 1 [file marinedrugs-17-00670-s001.pdf]

Tab.S1 Other alkaloids from the host ascidians

Compound	Structure	Host Ascidian	Activity	Ref.
Aplicyanin B<br>Aplicyanin D<br>Aplicyanin F	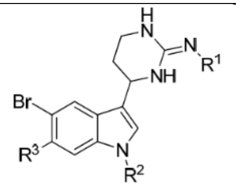<br>R_1=Ac,$R_{2,3}$=H Aplicyanin B<br>R_1=Ac,R_2=OMe, R_3=H Aplicyanin D<br>R_1=Ac,R_2=OMe, R_3=Br Aplicyanin F	*Aplidium cyaneum*	1.Potent antimitotic;<br>2.Cytotoxic against HT29, A549 and MDA-MB-231 cells.	[1]
Tanjungide A	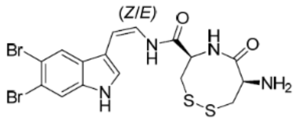	*Diazona* cf. *Formosa*	Cytotoxicity against A549, HT29 and MDA-MB-231 cells.	[2]
Pibocin A<br>Pibocin B	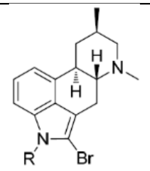<br>R=H Pibocin A<br>R=OMe PibocinB	*Eudistoma* sp.	Antimicrobial and cytotoxic effects against mouse Ehrlich carcinoma cells	[3]<br>[4]
3-bromofascaplysin	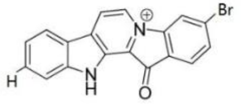	*Didemnum* sp.	Anticancer activity towards HL60, THP-1, HeLa, MDA-MB-231, DLD-1, SNU-C4 and SK-MEL-28 cells.	[5]<br>[6]

Tab.S1 *Cont.*

Eudistomidin B<br>Eudistomidin G	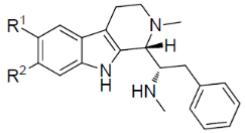<br>R_1 = Br, R_2 = H Eudistomidin B<br>R_1 = H, R_2 = Br Eudistomidin G	*Eudistoma glaucus*	Cytotoxic activity towards L1210 cell	[7]<br>[8]
Opacaline B<br>Opacaline C	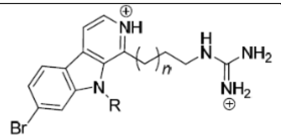<br>R = OH, n = 2 Opacaline B<br>R = H, n = 1 Opacaline C	*Pseudodistoma opacum*	Antimalarial activity against a chloroquine-resistant *Plasmodium falciparum*	[9]

Tab.S2 Other alkaloids from the ascidian-associated microbes

Compound	Structure	Origin microbe	Host Ascidian	Activity	Ref.
6-bromoindole-3-carbaldehyde	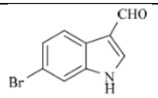	*Acinetobacter* sp.	*Stomozoa murrayi*	1.Antimicrobial activities to *Bacillus marinus* and *Vibrio campbellii;* 2. Inhibited the adhesion of cyprid larvae of *Balanus amphitrite.*	[10] [11]
Indole-3-carbaldehyde	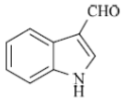				
Piericidin A1	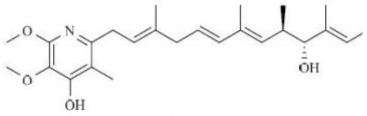	*Streptomyces* sp.	Unidentified ascidian fromIwayama	Inhibited the growth of RG-E1A-7 and Neuro-2a cells.	[12] [13] [14]
Piericidin A2	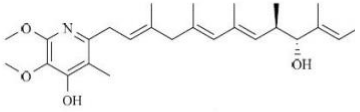				
Piericidin C7	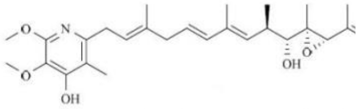				
Piericidin C8	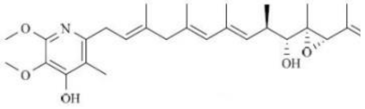				
1,6-dihydroxyphenazine	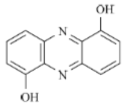	*Nocardiopsis dassonvillei*	*Botryllus schlosseri*	1.Antimicrobial activities to *Vibrioanguillarum* and *Vibrio parahaemolyticus*; 2. Lethal activity to *Artemia salina*; 3.Inhibit activity of Alpha-glucosidase.	[15] [16]

Tab.S2 *Cont.*

Bacillusamide B	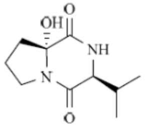	*Streptomyces* sp.	*Didemnum* sp.	Cytotoxic against HCT116, HepG2 and MCF7 cells.	[17] [18] [19]
Cyclo (6-OH-D-Pro-L-Phe)	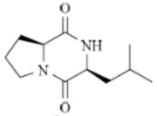				
	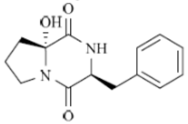				
Cyclo (L-Pro-L-Leu)					
Cyclo (L-Pro-L-Ile)	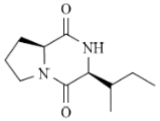				
Fumiquinazoline H	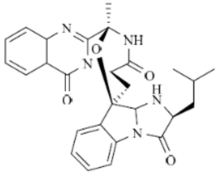	*Acremonium* sp.	*Ecteinascidia turbinate*	Antimicrobial activity against *Candida albicans.*	[20]
Fumiquinazoline I	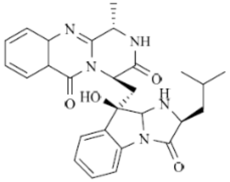				

Tab.S2 *Cont.*

(S)-6-(sec-butyl)-3-isopropylpyrazin-2(1H)-one	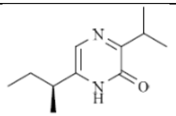				
(S)-3-(sec-butyl)-6-isopropylpyrazin-2(1H)-one	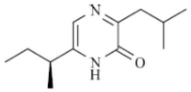				
(S)-6-(sec-butyl)-3-isobutylpyrazin-2(1H)-one	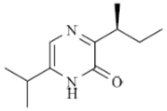	*Streptomyces* sp.	*Didemnum* sp.	Inhibited the growth of HCT116, HepG2 and MCF7 cells.	[21] [22]
(1H)-pyrazinones analogues deoxymutaaspergillic	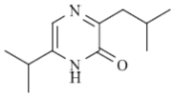				
Acid 3,6-diisobutyl-2(1H)-pyrazinone	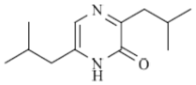				
3,6-disec-butyl-2(1H)-pyrazinone	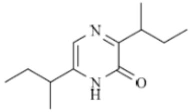				

Tab.S3 Other polypeptides from the host ascidians

Compound	Structure	Host Ascidian	Activity	Ref.
Cycloxazoline	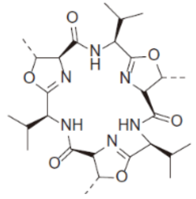	*Lissoclinum bistratum*	1. Inhibited HL60 cell accumulated in G2/M phase;<br>2. Cytotoxic against HL60, T24 and MRC5CV1 cells.	[23]
Lissoclinamide	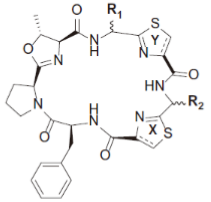	*Lissoclinum patella*	Inhibited the growth of human bladder carcinoma and fibroblast cell.	[24]
Ulithiacyclamide B	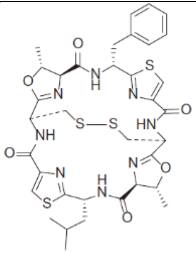	*Lissoclinum patella*	1. Cytotoxic against KB cells;<br>2.Inhibitor of Macrophage Scavenger Receptor.	[25]
Mollamide	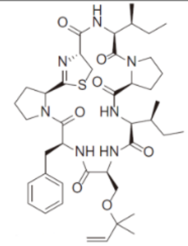	*Didemnum molle*	1.Cytotoxic against CV1, A549, HT29, and P388 cells;<br>2. RNA synthesis inhibitor.	[26]

Tab.S3 *Cont.*

Bistratamide E<br>Bistratamide F<br>Bistratamide G<br>Bistratamide H<br>Bistratamide I<br>Bistratamide J	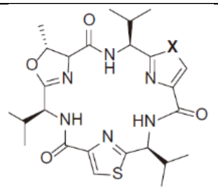<br>X= S Bistratamide E<br>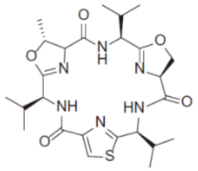<br>Bistratamide F<br>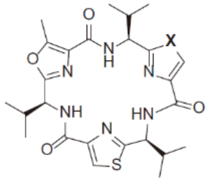<br>X= O Bistratamide G<br>X= S Bistratamide H<br>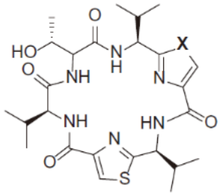<br>X = O Bistratamide I<br>X = S Bistratamide J	*L. bistratum.*	Cytotoxic against HCT 116 cells	[27]<br>[28]

Tab.S3 *Cont.*

Didmolamide A<br><br>DidmolamideB	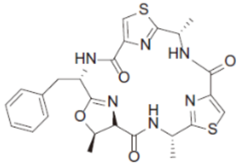<br>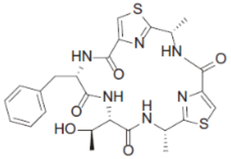	*Didemnum molle*	Cytotoxic against HT29, MEL28, and A549 cells.	[29]
Patellin 6	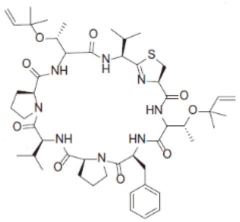	*Lissoclinum* sp.	1.Cytotoxic against CV1, A549, HT29, and P388 cells;<br>2. Inhibited topoisomerase II activation.	[30]
Virenamide A<br>Virenamides B<br>Virenamides C	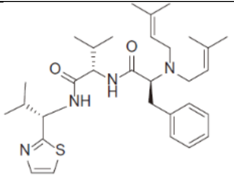<br>Virenamide A<br>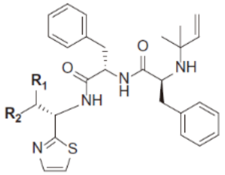<br>R_1= R_2= Me Virenamide B<br>R_1= H; R_2= Ph Virenamide C	*Diplosoma virens*	1. Against CV1, A549, P388and HT29 cells;<br>2. Inhibited topoisomerase II.	[31]

Tab.S4 Other Peptides from the ascidian-associated microbes

Compound	Structure	Origin microbe	Host Ascidian	Activity	Ref.
Peptidolipin B	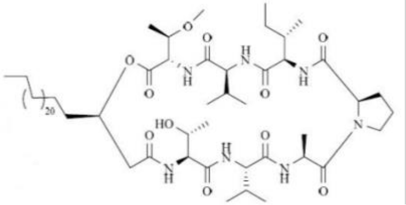	*Nocardia* sp.	*Trididemnum orbiculatum*	Inhibited methicillin-resistant *Staphylococcus aureus* and methicillin-sensitive *Staphylococcus aureus*.	[32] [33]
Peptidolipin E	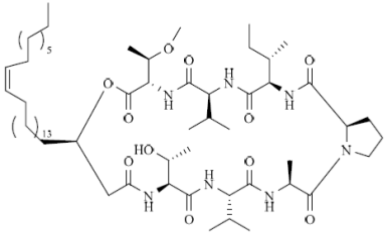				
Trichodermamide A	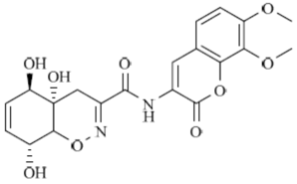	*Trichoderma virens*	*Didemnum molle*	1. Cytotoxic against HCT116 cell;<br>2.Antimicrobial activity against amphoterocin resistant *Candida albicans*, methacillin resistant *Staphylococcus aureus* and vancomycin resistant *Enterococcus faecium.*	[34] [35]
Trichodermamide B	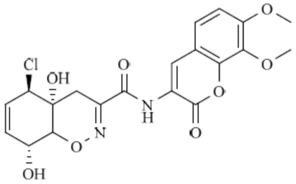				

Tab.S5 Other polyketides from the host ascidians

Compound	Structure	Host Ascidian	Activity	Ref.
Biselide A	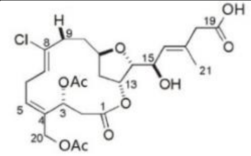	*Didemnidae* sp.	Inhibited the growth of MDA-MB-231 and NCI-H460 cells	[36]
Z-Rubrolide O	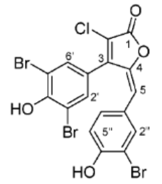	*Synoicumn* sp.	1. Inhibited superoxide production by human neutrophils;<br>2. Inhibited PMA-induced adhesion of neutrophils.	[37]
E-Rubrolide O	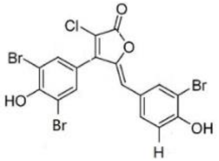			
Sphingosine 1a	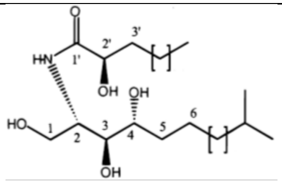	*Cystodytescf dellechiajei*	Inhibited the activity of phospholipase A2.	[38]
Mycalamide A	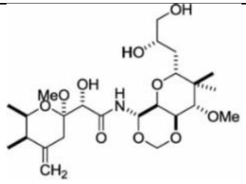	*Polysincraton* sp.	1. Prevented EGF-induced transformation induced apoptosis of JB6 Cl41 P+ cells<br>2. Inhabited the growth of HeLa cell.	[39]

Tab.S6 Other polyketides from the ascidian-associated microbes

Compound	Structure	Origin microbe	Host Ascidian	Activity	Ref.
Granaticin	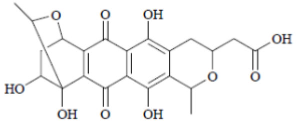				
Granatomycin D	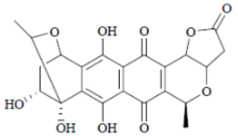	*Streptomyces* sp.	*Molgula manhattensis*	Against pathogens *Bacillus subtilis*, methicillin-sensitive *Staphylococcus aureus*, methicillin-resistant *Staphylococcus aureus*, and *Pseudomonas aeruginosa*.	[40] [41] [42]
Dihydrogranaticin B	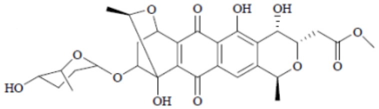				
Bisanthraquinone 1	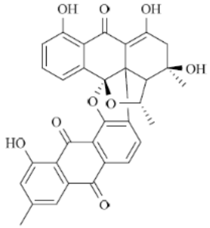	*Streptomyces* sp.	*Ecteinascidia turbinata*	1. Cytotoxic against HCT116 cell; 2. Antimicrobial activity against methicillin-resistant *Staphylococcus aureus* and vancomycin-resistant *Enterococcus faecalis*.	[43] [44]
Bisanthraquinone 2	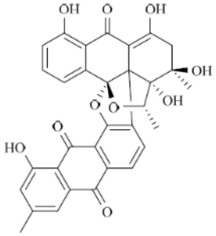				

Tab.S6 *Cont.*

Halomadurone C<br>Halomadurone D	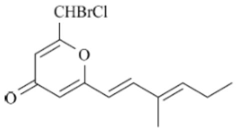<br>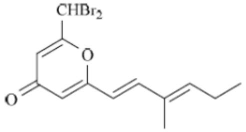	*Actinomadura* sp.	*Ecteinascidia turbinata*	Activated nuclear factor E2-related factor antioxidant response element (Nrf2-ARE).	[45]
Griseorhodin A	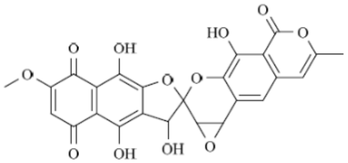	*Streptomyces* sp.	*Aplidium lenticulum*	Inhibited human telomerase and retroviral reverse transcriptase.	[46]
Ecteinamycin	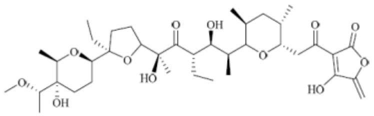	*Actinomadura* sp.	*Ecteinascidia turbinata*	Against microbial *Clostridium difficile* NAP1/B1/027.	[47]
Monodictyphenone	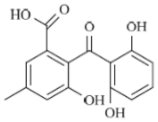	*Penicillium albobiverticillium*	Unidentified ascidian from Manado	Inhibited protein tyrosine phosphatase (PTP) 1B, T cell PTP (TCPTP), CD45 tyrosine phosphatase (CD45), and vaccinia H-1-related phosphatase (VHR).	[48]

Tab.S6 *Cont.*

Lomaiviticin A	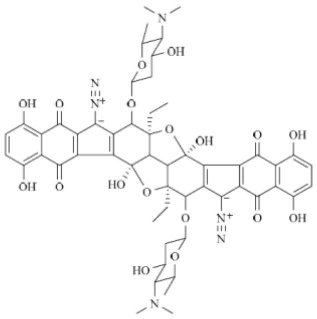	*Salinispora pacifica*	*Polysyncraton lithostrotum*	1. Potented DNA damaging;<br>2.Antimicrobial activities against *Staphylococcus aureus* and *Enterococcus faecium*;<br>3. Inhibited the growth of K562 cell.	[49]
Lomaiviticin B	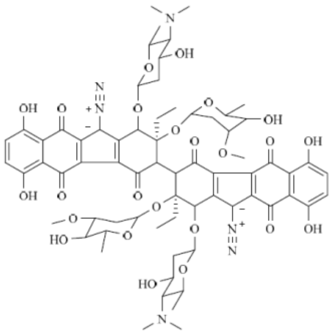				

Tab.S7 Other kinds of compounds from the host ascidians

Compound	Structure	Classification	Host Ascidian	Activity	Ref.
Prunolide A<br>Prunolide B<br>Prunolide C	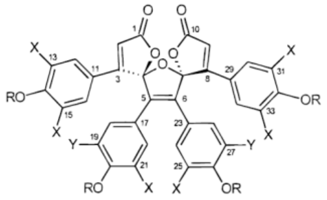<br>R=H, X=Br, Y=Br Prunolide A<br>R=H, X=Br, Y=H Prunolide B<br>R=H, X=H,Y=H Prunolide C	spiroketal	*Synoicum prunum*	Inhibited the growth of HeLa cell.	[50]
Rossinone A<br>Rossinone B	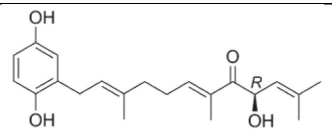<br>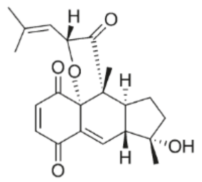	meroterpene	*Aplidium* sp.	1.Inhibited superoxide production;<br>2. Selective antiviral activity toward the DNA virus HSV-1 and the RNAvirusPV-1;<br>3. Antimicrobial activity against *Bacillus subtilis* and *Trichophyton mentagrophytes*;<br>4. Antiproliferative activity to P388 cells.	[51]<br>[52]
Aplidiasphingosine	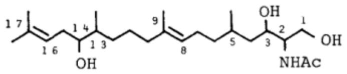	terpenoid	*Aplidium* sp.	Inhibited the growth of KB and L1210 cells	[53]

Tab.S7 *Cont.*

Halocyamines A	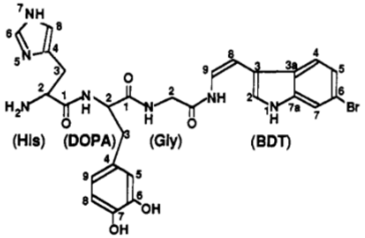	terpenoid	*Halocynthiaro retzi*	1.Inhibitory effects on the growth of *B. subtilis*, *B. megaterium*, *B.cereus* and the *Cryptococus neoformans*;<br>2. Cytotoxic against HepG2 and N18 cells.	[54]<br>[55]

Tab.S8 Other kinds of compounds from the ascidian-associated microbes

**Compound**	**Structure**	**classification**	**Origin microbe**	**Host Ascidian**	**Activity**	**Ref.**
Gifhornenolone A	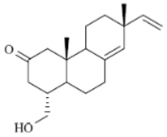	terpenoid	*Verrucosispora gifhornensis*	Unidentified ascidian from Hiroshima	Inhibited activity of androgen receptor.	[56]
Fuscoatrol A	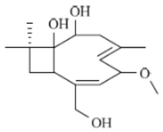	caryophyllene	*Humicola fuscoatra*	Kuril colonial ascidium	1. Against microbial *Staphylococcus aureus* and *Bacillus subtilis*; 2. Cytotoxic on the eggs development of *Strongylocentrotus intermedius.*	[57]
Verruculide A	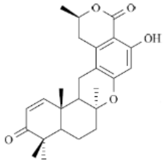					
Chrodrimanin A	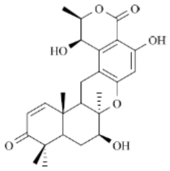	merosesquiterpenes	*Talaromyces verruculosus*	*Polycarpaaurata*	Inhibited the activity of protein tyrosine phosphatase 1B.	[58] [59] [60]
Chrodrimanin H	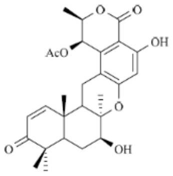					

Tab.S8 *Cont.*

5,8-epidioxy-23-methyl-(22E,24R)-ergosta-6,22-dien-3-ol	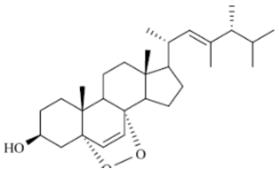	sterol	*Penicillium stoloniferum*	Unidentified ascidian from Qingdao	Inhibited the growth of P388 cells.	[61]
Penicillic acid	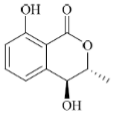	Furanones	*Aspergillus* sp.	*Eudistoma vannamei*	Cytotoxic against MDA-MB-435 and HCT8 cell lines.	[62]
2-hydroxy-6-(2O-hydroxy-3O-hydroxymethyl-5-methylphenoxy)-benzoic acid	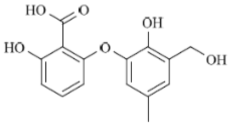	biphenyl ether	*Talaromyces albobiverticillius*	Unidentified ascidian from Manado	Inhibitory activities against protein tyrosine phosphatase 1B, T cell PTP and CD45 tyrosine phosphatase.	[63]
Oxepinamide A	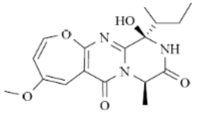	isocoumarin	*Acremonium* sp.	*Ectcinascidia turbinata*	Anti-inflammatory activity on ear edema of mouse.	[64]
Diterpene glycoside sordarin	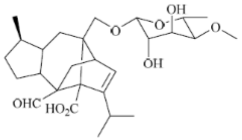	glycoside	*Talaromyces* sp.	Unidentified ascidian from Tweed Heads	Antifungal activity.	[65]

reference

1. Sisa, M., et al., *Total synthesis and antiproliferative activity screening of (+/-)-aplicyanins A, B and E and related analogues.* J Med Chem, 2009. **52**(20): p. 6217-23.
2. Murcia, C., et al., *Tanjungides A and B: new antitumoral bromoindole derived compounds from Diazona cf formosa. isolation and total synthesis.* Mar Drugs, 2014. **12**(2): p. 1116-30.
3. Liu, H., et al., *Total Syntheses of Festuclavine, Pyroclavine, Costaclavine, epi-Costaclavine, Pibocin A, 9-Deacetoxyfumigaclavine C, Fumigaclavine G, and Dihydrosetoclavine.* Org Lett, 2017. **19**(12): p. 3323-3326.
4. Makarieva, T.N., et al., *Pibocin B, the first N-O-methylindole marine alkaloid, a metabolite from the Far-Eastern ascidian Eudistoma species.* J Nat Prod, 2001. **64**(12): p. 1559-61.
5. Lyakhova, I.A., et al., *Antitumor Activity of Fascaplysin Derivatives on Glioblastoma Model In Vitro.* Bull Exp Biol Med, 2018. **164**(5): p. 666-672.
6. Segraves, N.L., et al., *Comparison of fascaplysin and related alkaloids: a study of structures, cytotoxicities, and sources.* J Nat Prod, 2004. **67**(5): p. 783-92.
7. Suzuki, T., T. Kubota, and J. Kobayashi, *Eudistomidins H-K, new beta-carboline alkaloids from the Okinawan marine tunicate Eudistoma glaucus.* Bioorg Med Chem Lett, 2011. **21**(14): p. 4220-3.
8. Murakami, Y., *[Chemistry of indoles: new reactivities of indole nucleus and its synthetic application].* Yakugaku Zasshi, 1999. **119**(1): p. 35-60.
9. Chan, S.T., et al., *Antimalarial beta-carbolines from the New Zealand ascidian Pseudodistoma opacum.* J Nat Prod, 2011. **74**(9): p. 1972-9.
10. McKay, M.J., et al., *1,2-bis(1H-indol-3-yl)ethane-1,2-dione, an indole alkaloid from the marine sponge Smenospongia sp.* J Nat Prod, 2002. **65**(4): p. 595-7.
11. Karimabad, M.N., et al., *Regulatory effects of the novel synthesized Indole-3-carbaldehyde on expression of cell cycle genes: A study on Cyclin D and P21 expression by acute promylocytic leukemia cell line (NB4).* Cell Mol Biol (Noisy-le-grand), 2017. **63**(5): p. 60-67.
12. Zhou, X. and W. Fenical, *The unique chemistry and biology of the piericidins.* J Antibiot (Tokyo), 2016. **69**(8): p. 582-93.
13. Morgan, J.M., et al., *Piericidin A1 Blocks Yersinia Ysc Type III Secretion System Needle Assembly.* mSphere, 2017. **2**(1).
14. Han, X., et al., *Geranylpyrrol A and Piericidin F from Streptomyces sp. CHQ-64 DeltardmF.* J Nat Prod, 2017. **80**(5): p. 1684-1687.
15. Zeyhle, P., et al., *A membrane-bound prenyltransferase catalyzes the O-prenylation of 1,6-dihydroxyphenazine in the marine bacterium Streptomyces sp. CNQ-509.* Chembiochem, 2014. **15**(16): p. 2385-92.
16. Zeyhle, P., et al., *Genome-based discovery of a novel membrane-bound 1,6-dihydroxyphenazine prenyltransferase from a marine actinomycete.* PLoS One, 2014. **9**(6): p. e99122.
17. Liu, Y.H., et al., *Diversity, community distribution and growth promotion activities of endophytes associated with halophyte Lycium ruthenicum Murr.* 3 Biotech, 2019. **9**(4): p. 144.
18. Jiang, S., et al., *Chalcomycins from Marine-Derived Streptomyces sp. and Their Antimicrobial Activities.* Mar Drugs, 2017. **15**(6).
19. Paulus, C., et al., *New natural products identified by combined genomics-metabolomics*

profiling of marine Streptomyces sp. MP131-18. Sci Rep, 2017. **7**: p. 42382.

20. Cheng, Z., et al., *Versiquinazolines A-K, Fumiquinazoline-Type Alkaloids from the Gorgonian-Derived Fungus Aspergillus versicolor LZD-14-1.* J Nat Prod, 2016. **79**(11): p. 2941-2952.
21. Buedenbender, L., et al., *HSQC-TOCSY Fingerprinting-Directed Discovery of Antiplasmodial Polyketides from the Marine Ascidian-Derived Streptomyces sp. (USC-16018).* Mar Drugs, 2018. **16**(6).
22. Harunari, E., et al., *Streptomyces hyaluromycini sp. nov., isolated from a tunicate (Molgula manhattensis).* J Antibiot (Tokyo), 2016. **69**(3): p. 159-63.
23. Watters, D.J., et al., *Accumulation of HL-60 leukemia cells in G2/M and inhibition of cytokinesis caused by two marine compounds, bistratene A and cycloxazoline.* Cancer Chemother Pharmacol, 1994. **33**(5): p. 399-409.
24. Degnan, B.M., et al., *New cyclic peptides with cytotoxic activity from the ascidian Lissoclinum patella.* J Med Chem, 1989. **32**(6): p. 1349-54.
25. Fu, X., et al., *New cyclic peptides from the ascidian Lissoclinum patella.* J Nat Prod, 1998. **61**(12): p. 1547-51.
26. Lu, Z., et al., *Thiazoline peptides and a tris-phenethyl urea from Didemnum molle with anti-HIV activity.* J Nat Prod, 2012. **75**(8): p. 1436-40.
27. Bertram, A., et al., *Synthesis of libraries of thiazole, oxazole and imidazole-based cyclic peptides from azole-based amino acids. A new synthetic approach to bistratamides and didmolamides.* Org Biomol Chem, 2007. **5**(10): p. 1541-53.
28. Ogino, J., et al., *Dendroamides, new cyclic hexapeptides from a blue-green alga. Multidrug-resistance reversing activity of dendroamide A.* J Nat Prod, 1996. **59**(6): p. 581-6.
29. Rudi, A., et al., *Didmolamide A and B, two new cyclic hexapeptides from the marine ascidian Didemnum molle.* J Nat Prod, 2003. **66**(4): p. 575-7.
30. Zhou, H., et al., *Patellin protein family functions in plant development and stress response.* J Plant Physiol, 2019. **234-235**: p. 94-97.
31. Carroll, A.R., et al., *Studies of Australian Ascidians. 5. Virenamides A-C, New Cytotoxic Linear Peptides from the Colonial Didemnid Ascidian Diplosoma virens.* J Org Chem, 1996. **61**(12): p. 4059-4061.
32. Wyche, T.P., et al., *Peptidolipins B-F, antibacterial lipopeptides from an ascidian-derived Nocardia sp.* J Nat Prod, 2012. **75**(4): p. 735-40.
33. Maget-Dana, R., et al., *Bacterial lipopeptides induce ion-conducting pores in planar bilayers.* Biochem Biophys Res Commun, 1985. **129**(3): p. 965-71.
34. Yang, Z., et al., *N-Me-trichodermamide B isolated from Penicillium janthinellum, with antioxidant properties through Nrf2-mediated signaling pathway.* Bioorg Med Chem, 2017. **25**(24): p. 6614-6622.
35. Lan, W.J., et al., *Five New Cytotoxic Metabolites from the Marine Fungus Neosartorya pseudofischeri.* Mar Drugs, 2016. **14**(1): p. 18.
36. Hayakawa, I., et al., *Total Synthesis of Biselide E, a Marine Polyketide.* Org Lett, 2017. **19**(20): p. 5713-5716.
37. Smitha, D., et al., *Rubrolide R: a new furanone metabolite from the ascidian Synoicum of the Indian Ocean.* Nat Prod Res, 2014. **28**(1): p. 12-7.
38. Ito, M., et al., *Sphingomyelins in four ascidians, Ciona intestinalis, Halocynthia roretzi,*

Halocynthia aurantium, and Styela clava. J Oleo Sci, 2009. **58**(9): p. 473-80.
39. Dyshlovoy, S.A., et al., *Mycalamide A shows cytotoxic properties and prevents EGF-induced neoplastic transformation through inhibition of nuclear factors.* Mar Drugs, 2012. **10**(6): p. 1212-24.
40. Jiang, B., et al., *6-Deoxy-13-hydroxy-8,11-dione-dihydrogranaticin B, an intermediate in granaticin biosynthesis, from Streptomyces sp. CPCC 200532.* J Nat Prod, 2014. **77**(9): p. 2130-3.
41. Gibson-Clay, G., S.R. Byrn, and P. Heinstein, *The interaction of granaticin with nucleic acids and pyruvate decarboxylase.* J Pharm Sci, 1982. **71**(4): p. 467-8.
42. Sung, A.A., S.M. Gromek, and M.J. Balunas, *Upregulation and Identification of Antibiotic Activity of a Marine-Derived Streptomyces sp. via Co-Cultures with Human Pathogens.* Mar Drugs, 2017. **15**(8).
43. Tian, W., et al., *Cytoskyrin C, an unusual asymmetric bisanthraquinone with cage-like skeleton from the endophytic fungus Diaporthe sp.* Fitoterapia, 2018. **128**: p. 253-257.
44. Rattanaburi, S., et al., *A new bisanthraquinone and cytotoxic xanthones from Cratoxylum cochinchinense.* Nat Prod Res, 2014. **28**(9): p. 606-10.
45. Wyche, T.P., et al., *Activation of the nuclear factor E2-related factor 2 pathway by novel natural products halomadurones A-D and a synthetic analogue.* Mar Drugs, 2013. **11**(12): p. 5089-99.
46. Lin, Z., et al., *Griseorhodins D-F, neuroactive intermediates and end products of post-PKS tailoring modification in Griseorhodin biosynthesis.* J Nat Prod, 2014. **77**(5): p. 1224-30.
47. Wyche, T.P., et al., *Chemical Genomics, Structure Elucidation, and in Vivo Studies of the Marine-Derived Anticlostridial Ecteinamycin.* ACS Chem Biol, 2017. **12**(9): p. 2287-2295.
48. Sumilat, D.A., et al., *A new biphenyl ether derivative produced by Indonesian ascidian-derived Penicillium albobiverticillium.* J Nat Med, 2017. **71**(4): p. 776-779.
49. Janso, J.E., et al., *Discovery of the lomaiviticin biosynthetic gene cluster in Salinispora pacifica.* Tetrahedron, 2014. **70**(27-28): p. 4156-4164.
50. Carroll, A.R., et al., *Prunolides A, B, and C: Novel Tetraphenolic Bis-Spiroketals from the Australian Ascidian Synoicum prunum.* J Org Chem, 1999. **64**(8): p. 2680-2682.
51. Appleton, D.R., et al., *Rossinones A and B, biologically active meroterpenoids from the Antarctic ascidian, Aplidium species.* J Org Chem, 2009. **74**(23): p. 9195-8.
52. Nunez-Pons, L., et al., *Natural products from Antarctic colonial ascidians of the genera Aplidium and Synoicum: variability and defensive role.* Mar Drugs, 2012. **10**(8): p. 1741-64.
53. Bharate, S.B., et al., *Kinase inhibitors of marine origin.* Chem Rev, 2013. **113**(8): p. 6761-815.
54. Azumi, K., H. Yokosawa, and S. Ishii, *Halocyamines: novel antimicrobial tetrapeptide-like substances isolated from the hemocytes of the solitary ascidian Halocynthia roretzi.* Biochemistry, 1990. **29**(1): p. 159-65.
55. Taylor, S.W., et al., *Oxidation of peptidyl 3,4-dihydroxyphenylalanine analogues: implications for the biosynthesis of tunichromes and related oligopeptides.* J Nat Prod, 1991. **54**(3): p. 918-22.
56. Shirai, M., et al., *Terpenoids produced by actinomycetes: isolation, structural elucidation and biosynthesis of new diterpenes, gifhornenolones A and B from Verrucosispora gifhornensis YM28-088.* J Antibiot (Tokyo), 2010. **63**(5): p. 245-50.
57. Takao, K., et al., *Total Synthesis of (+)-Cytosporolide A via a Biomimetic Hetero-Diels-Alder*

Reaction. J Am Chem Soc, 2015. **137**(50): p. 15971-7.

58. Yamazaki, H., et al., *Verruculides A and B, two new protein tyrosine phosphatase 1B inhibitors from an Indonesian ascidian-derived Penicillium verruculosum.* Bioorg Med Chem Lett, 2015. **25**(16): p. 3087-90.

59. Guo, J., et al., *Tafuketide, a phylogeny-guided discovery of a new polyketide from Talaromyces funiculosus Salicorn 58.* Appl Microbiol Biotechnol, 2016. **100**(12): p. 5323-38.

60. Bai, T., et al., *Elucidation and Heterologous Reconstitution of Chrodrimanin B Biosynthesis.* Org Lett, 2018. **20**(23): p. 7504-7508.

61. Yang, Z., et al., *Molecular characterization of a new gammapartitivirus isolated from the citrus-pathogenic fungus Penicillium digitatum.* Arch Virol, 2018. **163**(11): p. 3185-3189.

62. Nguyen, H.T., et al., *Antibacterial activities of penicillic acid isolated from Aspergillus persii against various plant pathogenic bacteria.* Lett Appl Microbiol, 2016. **62**(6): p. 488-93.

63. Brophy, C.J., J. Moxham, and M. Green, *The effect of aminophylline on respiratory muscle contractility.* Prax Klin Pneumol, 1988. **42 Suppl 2**: p. 839-41.

64. Ebada, S.S., et al., *Psychrophilin E, a new cyclotripeptide, from co-fermentation of two marine alga-derived fungi of the genus Aspergillus.* Nat Prod Res, 2014. **28**(11): p. 776-81.

65. Chang, Y.C., et al., *Diterpene glycosides and polyketides from Xylotumulus gibbisporus.* J Nat Prod, 2014. **77**(4): p. 751-7.
